# Barriers That Obstruct Return to Work After Coronary Bypass Surgery: A Qualitative Study

**DOI:** 10.1007/s10926-020-09919-6

**Published:** 2020-08-16

**Authors:** Fredrike Blokzijl, Marisa Onrust, Willem Dieperink, Frederik Keus, Iwan C. C. van der Horst, Wolter Paans, Massimo A. Mariani, Michiel F. Reneman

**Affiliations:** 1 Department of Cardiothoracic Surgery, University Medical Center Groningen, University of Groningen, Hanzeplein 1, PO Box 30001, 9700 RB Groningen, The Netherlands; 2 Department of Critical Care, University Medical Center Groningen, University of Groningen, Groningen, The Netherlands; 3 Department of Intensive Care, Maastricht University Medical Center+, University of Maastricht, Maastricht, The Netherlands; 4 Research Group Nursing Diagnostics, Hanze University of Applied Sciences, Groningen, The Netherlands; 5 Department of Rehabilitation Medicine, University Medical Center Groningen, University of Groningen, Groningen, The Netherlands

**Keywords:** Return to work, Coronary artery bypass, Absenteeism, Cardiac rehabilitation

## Abstract

*Purpose* Coronary artery bypass grafting is the most frequently performed cardiac surgical procedure. Despite its benefits on survival and quality of life, it is associated with a considerable financial burden on society including sick leave. Our study aimed to explore the barriers that obstruct return to work after coronary artery bypass grafting. *Methods* We performed a qualitative study with in-depth interviewing of patients 6 months after their surgery. We included ten working patients and interviewed them and their spouses at home. The interviews were transcribed and two investigators independently searched the transcriptions for barriers that had obstructed return to work. *Results* Based on the interviews we were able to distinguish four main groups of barriers: ‘personal’, ‘healthcare’, ‘work’ and ‘law & regulation.’ The personal barriers were subgrouped in affective, physical, cognitive, social and individually determined factors. *Conclusion* In a qualitative study we showed that personal barriers as well as barriers regarding healthcare, work and law & regulation, were perceived by patients as important factors obstructing return to work after coronary artery bypass grafting. To overcome the identified barriers, the process of return to work could preferably be initiated during the hospital phase, started during cardiac rehabilitation, and coordinated by a case-managing professional.

**Electronic supplementary material:**

The online version of this article (10.1007/s10926-020-09919-6) contains supplementary material, which is available to authorized users.

## Introduction

In the Netherlands, over 15,000 cardiac surgeries are performed every year [1]. The main reasons to offer cardiac surgery, including coronary artery bypass grafting (CABG) are to improve survival and quality of life [2]. A relevant number of patients are at working age when undergoing coronary bypass and return to work (RTW) is an important goal during recovery [3, 4]. Because ischemic heart disease, including coronary artery disease, can be life-threatening, anxiety and uncertainty have been suggested as barriers for RTW, not only for the patient but also for the occupational physician [3, 4]. Guidelines of the Dutch society of occupational physicians describe how to guide employees with ischemic heart disease during the process of resuming their work [4]. These guidelines are based on internationally conducted studies and recommend to resume work during cardiac rehabilitation (CR) to overcome possible barriers of fear and anxiety. The guidelines suggest partial or fully RTW within approximately six weeks for patients with coronary artery disease, including patients after CABG, provided that postoperative biological recovery was uncomplicated [4].

Only a few studies on return to work after CABG have been conducted. RTW is observed between 8 and 13 weeks after surgery in two studies with CABG patients not participating in cardiac rehabilitation-programs [5, 6] while another study reported 64% of patients fully resuming work within 6 months after cardiac rehabilitation [7]. There seems to be a notable discrepancy between guidelines recommendations on RTW after CABG and literature reports, although evidence is scarce. Younger age [6, 8, 9], higher job satisfaction [5], positive occupational expectations [7, 10] and absence of diabetes and myocardial damage [6, 8, 11] have been suggested to facilitate quicker RTW but may not be considered comprehensive, because they are all individually determined, internal factors. In RTW models, such as the case-management ecological model, external factors, factors outside the individual, i.e. work-related and other factors influencing the disability process are also present [12]. While this model was developed for the case management of disability due to low back pain and has been applied in other medical conditions, it has not been used in patients after CABG.

In-depth interviewing of patients after CABG and their spouses may enrich our knowledge on the process of RTW after CABG from the patient perspective including the facilitators and barriers influencing this process. The aim of our study was to identify barriers that obstruct return to work of patients after CABG.

## Methods

### Study Design

The study is reported according to the Standards for Reporting Qualitative Research (SRQR) [13]. A qualitative study design was applied to evaluate barriers that obstruct return to work in patients after CABG. Through in-depth interviewing of patients and their partners we explored personal experiences [14]. An essential characteristic of the data collection by in-depth interviewing is that critical issues identified in one interview are used to refine questions and topics in the next interview. This design of inductive inference provides the opportunity to elaborate each issue with each subsequent interview [14].

### Participants

Participants were selected based on distinct inclusion criteria: all patients underwent elective, isolated CABG, were living with a spouse, and had paid work before surgery. To ensure that other severe comorbidities or a complicated recovery did not affect the process of RTW, we excluded patients with the following preoperative comorbidities: ejection fraction < 30%, stroke, psychiatric illnesses, chronic obstructive pulmonary disease (COPD) GOLD III-IV or renal disease (a reduced renal function prior to surgery with an estimated Glomerular Filtration Rate (eGFR) < 60 ml/min/1.73 m^2^ [15]. We also excluded patients who participated in the Heart-ROCQ study, a randomized trial on an extended CR-program before and after cardiac surgery [16].

### Sample and Setting

All interviews were held at the participants’ homes between 6 and 7 months after their CABG surgery in line with an expected full recovery. Based on current literature, we defined that data saturation would be reached when in three subsequent interviews, no new additional themes emerged [17].

### Data Collection

The interviews were semi-structured, using an interview guide which was divided into different topics based on recent literature [13, 17] (Appendix 1 in Electronic supplementary Material). The interviewer can use an interview guide as a list of topics and open-ended questions; the guide was not used as a questionnaire but rather as a memory aide during the interview [14]. To start, some baseline questions were asked to identify narratives of people's lives (i.e. type of work, education level, working hours). All interviews were conducted by one of the researchers (FB) who works at the university hospital as a nurse practitioner and PhD-student at the department of cardiothoracic surgery. All interviews were audiotaped with permission from the participants.

### Data Analysis

After verbatim transcriptions, two researchers (FB and MO) independently performed data analyses following the thematic content approach, which focuses on identifying, analyzing and interpreting patterns of meaning within qualitative data [18]. All transcriptions were examined to create one large preliminary list of barriers obstructing return to work as derived from the in-depth interviews [19]. Both researchers independently created items and after inductive and axial coding using consensus techniques, more collectively shared underlying concepts were analysed leading to a codebook utilized by the qualitative analysis software package ATLAS.ti. (version 8, ATLAS.ti Scientific Software Development, Berlin) [20]. Finally, higher-level grouping was guided by the case-management ecological model [12]. The frequency of the individual barriers was assessed using quantitative techniques by determining how frequent each item was mentioned. The frequency of all mentioned barriers was only used in a semi-quantitative approach to guide emphasis in the description of the results section.

### Ethical Considerations

The institution’s ethical committee approved the protocol (reference number 2019/075) and waived the need for formal evaluation according to the Dutch Law on Scientific Medical Research with Humans. All participants were approached by telephone for their participation in this study and formal written informed consent was obtained prior to the interview. To ensure anonymity, all data that may plausibly identify any of the participants were eliminated from the transcripts.

## Results

All interviews were held between March and June 2019. No additional themes emerged after interviewing participant eight, nine and ten so data saturation was reached after ten interviews. Baseline characteristics of the participants are listed in Table 1. Five participants had fully returned to work; two after 2 months and three within 4–6 months. The other five participants were still in the process of returning to work guided by an occupational physician. Two participants were self-employed. One participant reported that during hospital admittance, a nurse practitioner in the hospital had discussed the expectations regarding RTW with the participant. All patients had participated in a multidisciplinary CR-program after CABG, either a phase I program (inpatient CR-program) (n = 2) or a phase II program (an early post-discharge outpatient CR-program (n = 8)). Nine participants reported that the process of RTW had started after having finished the rehabilitation program, and seven participants reported their impression that there was no communication between the physicians involved.

**Table 1:** Characteristics of participants

Participant	Age (years)	Gender	Education-level	Occupation (collar)	Employment	Contract hours
1	57	Male	High	White	Employed	32
2	49	Male	High	White	Employed	40
3	62	Male	Low	Blue	Employed	40
4	61	Male	Medium	White	Employed	40
5	56	Male	High	White	Employed	36
6	53	Male	High	White	Self-employed	> 40
7	56	Male	Medium	Pink (service industry)	Employed	40
8	63	Male	Medium	White	Self-employed	> 40
9	59	Female	High	White	Employed	40
10	54	Male	Low	Blue	Employed	40

After verbatim transcription and analyses of the data, four main groups of barriers were defined: ‘personal’, ‘healthcare’ ‘work’ and ‘law and regulation’. The personal barriers were divided into five subgroups: 'affective', ‘physical’, ‘individually determined’, ‘social’ and ‘cognitive’. All barriers and associated items are listed in Table 2.

**Table 2:** Barriers that obstruct return to work in patients after CABG

Personal	132 (55%)
*Affective*	57
Loss of self-confidence	12
Anxiety	10
Feeling bad due to physical limitations	9
Not being able to handle a lot of fuss	9
Feeling of being out of balance	5
Fear of becoming physically active again	4
Post-traumatic stress	3
Awareness of suddenly being a heart patient	3
Grief/very emotional	2
*Physical*	43
Fatigue	17
Physical complaints due to other health issues	16
Loss of condition	8
Chest pain	2
*Individually determined*	13
Feeling obliged to resume work	5
No attention for others (focusing on yourself)	3
Change in personality due to illness	2
Feeling superfluous at work during reintegration	2
Not being able to let go of work during reintegration	1
*Social*	12
Family experiencing stress	7
Social environment advising to take it easy	5
*Cognitive*	*7*
Memory loss	4
Concentration disorder	3
Healthcare	63 (26%)
No advice concerning RTW	24
No guidance/follow-up after cardiac rehabilitation	15
Discouraging RTW	14
Employment consultant available but not involved	3
Physicians conflicting opinions concerning RTW	3
Pressure by occupational physician to RTW	3
Leaflet concerning RTW not applicable	1
Work	38 (16%)
Factors causing work stress	18
Communication with supervisor	13
High costs to hire a replacement (self-employed)	3
Interaction with colleagues	3
Sole employee in the own company	1
Law and regulation	6 (3%)
Limited insurance when ill (self-employed)	3
Temporary contract: job loss due to illness	2
Uncertainty about income	1

Within the personal barrier, the subgroup concerning affective factors was mentioned most frequent, i.e. loss of self-confidence, anxiety, bad feeling due to physical complaints & limitations, and being unable to handle a lot of fuss. Participants for example mentioned: “… the fear that you visit the cardiologist in the morning and are put in a wheelchair immediately and admitted because you are a ticking time bomb although you felt great, that realization of being a heart patient all of a sudden was very emotional …”[P (participant) 1]. “…The fact that you simply could not have made it and luckily that is not the case but well, the thought was there because it is your heart…” [R (relative) 5]. One of the partners described the uncertainty as her husband being out of balance: “…because being healthy is a balance and if you are physically out of balance, then this also impacts mentally and right now, his balance is lost in everything…” [R.5]. Among the physical barriers fatigue was often mentioned. Physical complaints due to other health issues and loss of condition were relevant as well. One participant mentioned: “…then I arrive at work and I am very busy, thousand things to do and when I go home I am exhausted while there is still plenty to do at home but I am not capable of doing anything after work…”[P.4]. Among the ‘individually determined’ factors a number of participants mentioned that they were not able to let go of work during their illness: “…I work as a car salesman and during my rehabilitation I was talking with the social worker and she said to me ‘you are so busy with your work that you are trying to sell me a car just now’, and I thought she is completely right…”[P.7]. Among the social factors family experiencing stress or family members advising to take it easy were mentioned. For example: “…it was difficult to see that my wife was so busy with managing everything because on the one hand I needed time for myself to recover but on the other hand I also thought, I am needed at home and in my own company…”[P.6]. During RTW some of the participants experience cognitive problems such as memory loss or concentration disorders: “…My memory isn't great either. My reactions are slower and when I am working and someone comes in to ask me a question, I really have to concentrate and realize what I was doing before the disturbance…”[P.2].

Among the barriers concerning healthcare, patients mentioned the lack of advice concerning RTW, professionals discouraging RTW, and no follow-up after cardiac rehabilitation, as the most critical barriers in the process of RTW. Participants for example mentioned: “… when I asked my cardiologist about RTW he said to me that work did not interest him, my health was his priority, not work…” [P.3]. “… the occupational physician advised me to take it easy, but I did not know exactly what to do with this advice…” [P.4]. “… during cardiac rehabilitation there was an informative meeting in the hospital for cardiac patients, and we saw a short movie of a man who wanted to RTW after surgery, but that he did not succeed at all. That was not an uplifting video because my wife and I thought I would go back to work pretty soon…” [P.1]. Another participant: “… because actually after the rehabilitation then suddenly there is nothing left. Yes, then there is your job—you have to pick up all vocational and social activities again but there is no guidance or whatsoever…” [P.2].

In the work-related barrier, factors causing work stress (i.e. excessive workload, busy workplace, understaffed) and the communication with the supervisor (i.e. difficult relationship) were mentioned most frequently.

## Discussion

This qualitative research showed that personal as well as healthcare-, work- and law & regulation-related factors, are all barriers in returning to work after CABG. Personal barriers concerning affective or physical complaints were most frequently mentioned during the interviews. Remarkably, many participants were dealing with feelings of anxiety, loss of self-confidence and fatigue during RTW, although their CABG was uncomplicated from a medical perspective.

Other recent studies also revealed that affective factors play an essential role in RTW after a cardiac event and suggest to identify this as soon as possible to allow for targeted psychosocial care [7, 10, 11]. We did not identify other studies confirming our findings on physical barriers such as fatigue that impedes resumption of work after CABG. Previous studies though, focused on patients with coronary artery disease, including also patients after less invasive procedures such as percutaneous coronary interventions or patients treated by medication only. These patients deviate severely from patients after coronary bypass as CABG is a major invasive procedure with impact on the entire body both physically (i.e. a sternal wound, postoperative anemia) as well as mentally, likely leading to prolonged recovery. The invasiveness of the treatment likely explains the long-term complaints of fatigue and feelings of anxiety. Because CR-participants after CABG deviate from other CR-participants in general, further research may possibly lead to adapted guidelines for patients after cardiac surgery. The previously mentioned guidelines suggest RTW within six weeks but based on recent studies [5–7] and on the findings in our study, this may not be feasible for patients after coronary bypass.

Other findings include the healthcare-related barriers on RTW after CABG. Remarkably, there is either complete absence or contradictory advice and guidance from the healthcare professionals. Many participants mentioned that surgeons, cardiologists, general practitioners, or occupational physicians did not advise them concerning RTW, or alternatively, the given advice was discouraging or unclear (e.g. the advice to ‘take it easy’). This pattern of contradictory advice or lack of advice was previously recognized in a study on RTW for patients after cholecystectomy [21]. No or unclear advice may negatively interact with the mentioned feelings of anxiety and uncertainty among patients and their spouses. Also, participants indicated that there appeared to be no communication between the physicians involved. All participants joined a CR-program following international guidelines for patients with CAD, including patients after coronary bypass [22–24]. An essential goal of CR is to optimize participation in society regarding different aspects of daily life, such as domestic, occupational, and recreational activities [23, 25]. While participants were positive about their CR-program, participants also mentioned that the lack of follow-up after CR induced feelings of uncertainty about their activities at home and in resuming work. These feelings of uncertainty became even stronger because participants were advised to start the process of RTW only *after* completing CR, which is in contrast with guideline recommendations. The only participants returning to work during CR and within 2 months after surgery were the participants who were self-employed. Returning to work during CR requires coordination between the occupational physician and the cardiac rehabilitation team which is in line with the suggestions for improvement mentioned in the Dutch rehabilitation guidelines [3]. A case-managing professional as a central contact is insufficiently imbedded in current practice resulting in inadequately aligned treatment- and RTW-plans [3].

After the interviews many participants reported that they felt the importance to improve the process of RTW after CABG, which was their incentive to participate in this study. We fully agree with the participants as there is a lot to improve. RTW itself can improve quality of life and economic security from both the individual [26] and the societal perspective; a quicker RTW can lead to reductions in work stress, sick-leave and substantial savings in indirect costs. Three recent reviews on RTW for workers with musculoskeletal pain-related conditions and on workers with coronary heart disease have suggested that long term absence is significantly reduced by multi-domain interventions such as healthcare provision, coordination between healthcare providers and the workplace, and work accommodation components [11, 27, 28]. Suggested practical actions could be: identification of negative chronic conditions that cause work-related stress, individual RTW training, contacting and discussing the RTW strategy with the employer and, organization of financial security [11]. Multi-domain interventions may also be of added value in the process of RTW after CABG. Feelings of anxiety and uncertainty can possibly be reduced or overcome by an earlier start of the process of RTW causing positive feedback (self-efficacy) or by proper counseling by healthcare professionals.

This study has several limitations. We focused on individual aspects using a qualitative design and results should therefore be considered in an explorative perspective. Further quantitative studies are necessary on RTW after coronary bypass to demonstrate the importance of the identified barriers. We only included patients with a spouse, with paid work, and without severe comorbidities limiting generalizability. Also, we excluded patients with complicated recovery, and our study population included only one woman and nine men. Although we did not use member checking or participant feedback to see whether the participants recognized the results, it is reassuring that most factors reflect the items from the case-management ecological model and another Dutch study on barriers influencing RTW after surgery [21, 29]. The results may be different in countries without a social security system, so that factors influencing RTW differ as well in these countries. This study focused on perspectives of patients and their spouses. Future studies should also focus on perspectives of supervisors and coworkers, as well as healthcare professionals.

## Conclusion

Several barriers play a role in the process of return to work after coronary bypass. Affective and physical barriers and the absence or contradictory guidance from the healthcare professionals involved were mentioned most often. As guidelines suggest, the process of RTW could preferably be initiated during the hospital phase, started during CR, and coordinated by a case-managing professional to overcome the barriers of RTW after CABG.

## Electronic supplementary material

Below is the link to the electronic supplementary material.

Supplementary file1 (DOCX 19 kb)
