# Supplementary material for: Barriers That Obstruct Return to Work After Coronary Bypass Surgery: A Qualitative Study
Source: J Occup Rehabil. 2020 Aug 16;31(2):316–22. doi: 10.1007/s10926-020-09919-6 (PMC8172483; doi:10.1007/s10926-020-09919-6)
Supplement: Supplementary file 1 — Supplementary file1 (DOCX 19 kb) [file 10926_2020_9919_MOESM1_ESM.docx]

**Appendix I – Interview guide**

*Introduction*

1. What kind of work do you have?
2. On average how many hours did you work before surgery?
3. Can you tell me something more about the work you do?

Next questions classified according to the conceptual model

*Questions related to work*

1. How do you experience your work?
2. Do you have to deal with stressors at work? Can you tell me something more about (possible) stressors?
3. How do you experience the contacts with your supervisor and / or colleagues?

*Questions related to the healthcare system*

1. Did you participate in a cardiac rehabilitation program after the operation? How did you experience this?
2. What were your experiences with the general practitioner/cardiologist/surgeon with regard to resuming work?
3. How did you experience the guidance for resuming work?

*Questions concerning your health*

1. Have you noticed any physical complaints after your heart surgery?
2. Have you experienced any psychosocial problems after surgery?
3. How did you experience the support of your family and friends?

*Questions regarding insurance (if applicable)*

1. Are you insured in the event of illness (self-employed)?
2. If you are not working right now, are you worried about your income or costs?

*Final questions*

1. Looking back on the period after your heart surgery, are there any aspects in the process of RTW that you think could be improved?
2. Which barriers may have an effect on resuming work after coronary bypass?
